# Skeletal cryptococcosis from 1977 to 2013

**DOI:** 10.3389/fmicb.2014.00740

**Published:** 2015-01-14

**Authors:** Heng-Xing Zhou, Lu Lu, Tianci Chu, Tianyi Wang, Daigui Cao, Fuyuan Li, Guangzhi Ning, Shiqing Feng

**Affiliations:** Department of Orthopaedics, Tianjin Medical University General HospitalTianjin, China

**Keywords:** skeletal cryptococcosis, *Cryptococcus neoformans*, immune status, underlying disease, dissemination

## Abstract

Skeletal cryptococcosis, an aspect of disseminated cryptococcal disease or isolated skeletal cryptococcal infection, is a rare but treatable disease. However, limited information is available regarding its clinical features, treatment, and prognosis. This systematic review examined all cases published between April 1977 and May 2013 with regard to the factors associated with this disease, including patient sex, age, and epidemiological history; affected sites; clinical symptoms; underlying diseases; laboratory tests; radiological manifestations; and delays in diagnosis, treatment, follow-up assessments, and outcomes. We found that immune abnormality is a risk factor but does not predict mortality; these observations are due to recent *Cryptococcus neoformans* var *gattii* (CNVG) outbreaks (Chaturvedi and Chaturvedi, 2011). Dissemination was irrespective of immune status and required combination therapy, and dissemination carried a worse prognosis. Therefore, a database of skeletal cryptococcosis cases should be created.

## Introduction

Cryptococcosis, formerly known as torulosis, European blastomycosis, or Busse-Buschke disease, is caused by *Cryptococcus neoformans* (*C neoformans*). This species was first isolated from peach juice by Sanfelice in 1894 (Mitchell and Perfect, 1995; Jain et al., 2013). Cryptococcus is a spherical-to-oval, encapsulated, yeast-like fungus that is widespread in spoiled milk, soil, and bird droppings, especially pigeon excreta. *C neoformans* can be divided into *Cryptococcus neoformans* var neoformans (CNVN) and *Cryptococcus neoformans* var *gattii* (CNVG), both of which are pathogens in humans and animals. They were not considered different varieties until 1970, when CNVG was officially suggested as a new species based on mounting evidence discovered since the first CNVG report in 1896 (Speed and Dunt, 1995; Chaturvedi and Chaturvedi, 2011; Harris et al., 2011). In addition, an on-going CNVG outbreak originated in 1999 and reappeared in 2004 (Chaturvedi and Chaturvedi, 2011). The most affected organs are the lungs and central nervous system, but virtually any organ (e.g., the skin, joints, eyes, urinary tract, liver, prostate, myocardium, muscles, kidneys, and bone) can be involved through lymphangitic and hematogenous spread after inhaling fungal propagules. Skeletal cryptococcosis is rare. Furthermore, this disease can be divided into two types: skeletal cryptococcosis, which is an aspect of disseminated cryptococcosis, and primary skeletal cryptococcosis, which does not involve other tissues (Chleboun and Nade, 1977; Behrman et al., 1990; Wood and Miedzinski, 1996). Due to its low morbidity rate, little is known about this disease. Moreover, its basic clinical features, treatment, and prognosis have long perplexed clinicians. Serious consequences, including death, have occurred in certain patients (Singh and Xess, 2010). Therefore, a systematical retrospective analysis of skeletal cryptococcosis is crucial for understanding this disease. Unfortunately, however, almost all of the current studies regarding this disease have been presented as case reports (Ramkillawan et al., 2013; Zhou et al., 2013), which provides little and indirect insight for understanding of skeletal cryptococcosis. Thus, a systematic, retrospective analysis of all published cases of skeletal cryptococcosis reported between April 1977 and the present time was conducted to clarify its clinical features, treatments, and prognoses—all of which are critical issues for fully understanding this disease. Importantly, treatment and prognostic suggestions based on the analysis are provided.

## Materials and methods

### Search strategy and selection criteria

A systematic online search was performed for cases reported over a 36-year period from April 1977 to the present time using PubMed, Medline, EBSCO, SpringerLink, Ovid, Highwire, ProQuest, and Wiley InterScience. We applied the following algorithm in both the medical subject heading (MeSH) and the search field. The MeSH terms “case reports” and “review” were combined with “Cryptococcus,” “*Cryptococcus neoformans*,” “osteomyelitis,” or “immunocompromised host,” and these MeSH terms were exploded when appropriate. Search terms such as “cryptococcosis,” “skeletal,” “bone,” “joints,” “skull,” “arthritis,” “disseminated,” or “immunocompetent” were also combined with the MeSH terms to increase the number of relevant articles retrieved. Google Scholar was also searched, and the citations in each article were reviewed to identify additional references that were not retrieved during the primary search. Language restrictions were not applied, and two researchers independently conducted all searches.

“Disseminated skeletal cryptococcosis” was defined as an infection that involves two or more non-contiguous bone sites or an infection that involves one bone site associated with extra-skeletal sites; patients with soft tissue collections or abscesses adjacent to the involved bone were excluded (Wood and Miedzinski, 1996). “Classically immunodeficient conditions” were considered to include corticosteroid use, HIV infection, interleukin-2 deficiency, and T cell defects (Speed and Dunt, 1995; Yu et al., 2012). Patients with other underlying diseases that affect immune function, such as diabetes mellitus, tuberculosis and connective tissue disorders, were considered relatively immunocompromised. “Osteomyelitis” was defined based on a positive radiograph, bone scan, or histopathology (Harris et al., 2011). “Relapse” was defined as the recurrence of symptoms at the previous disease site and the rediscovery of viable cryptococci from a previously checked sterile body site after successful primary therapy (Perfect et al., 2010).

### Data collection and analysis

The following information was retrospectively reviewed: patient sex, age, and epidemiological history; involved sites; clinical symptoms; underlying diseases; laboratory tests; radiological manifestations; and delays in diagnosis, treatment, follow-up assessment, and outcomes. Outcome was recorded as either response (i.e., resolution or improvement of all signs and symptoms, including microbiological and serological abnormalities and radiographic changes due to infection) or failure (i.e., deterioration of the patient's condition based on clinical features and radiographic abnormalities, ultimately resulting in death) (Kontoyiannis et al., 2001).

All cases were epidemiologically and clinically analyzed. The hosts were categorized as patients with immune abnormalities (including classically immunodeficient and relatively immunocompromised status) or as immunocompetent.

### Statistical analyses

Statistical analyses were performed using IBM SPSS 18.0 (IBM Corporation, Armonk, NY, USA). All continuous data were expressed as means ± standard deviations (means ± SDs), and comparisons were performed using One-Way ANOVAs. Categorical variables were compared using the χ^2^-test. All tests were two-tailed, and *p* < 0.05 were considered significant.

## Results

In total, 80 articles (including one written in Spanish [case 50] and 79 written in English) that described 89 patients were collected and analyzed retrospectively (Table 1). Case 25 recorded only the affected site (orbit) and treatment (amphotericin [AMB] and ketoconazole); thus, this case was removed from the analysis due to unknown immune status.

**Table 1:** **Summary of 89 cases of cryptococcosis of the bones and joints**.

**Case no./References**	**Age/Sex**	**Bone or joint**	**ESR**	**Osteomyelitis**	**Dissemination**	**Underlying diseases**	**Treatment**	**Outcome**	**Follow up**
1/ Chleboun and Nade, 1977	43/M	Left tibia	18	No	No	Sarcoidosis	Surgery	Failure	2 years
2/ Chleboun and Nade, 1977	40/F	Left ulna	44	No	No	Sarcoidosis	Surgery	Response	15 months
3/ Chleboun and Nade, 1977	68/M	Left scapula	92	Yes	No	Renal cyst	Surgery	Response	4 years
							AMB 1000 mg		
4/ Chleboun and Nade, 1977	15/M	Left humerus	NA	No	No	Normal	AMB 395 mg	Failure	2 years
5/ Bryan, 1977	26/M	T5	NA	Yes	No	Normal	AMB 2361 mg	Response	1 years
6/ Poliner et al., 1979	1.3/M	C2, C3	Normal	Yes	No	Normal	Surgery	Response	16 months
							AMB 34 mg+5-FC 73 g		
7/ Meredith et al., 1979	36/M	Right 2nd, 3rd ribs, C6, C7	NA	Yes	Skin	Normal	AMB+5-FC	Response	NA
8/ Fialk et al., 1981	36/M	Left humerus, right femur, left iliac wing	NA	No	Lung, skin	Normal	Surgery+AMB 2 g	Response	15 years
9/ Fialk et al., 1981	9/M	Left tibia	Normal	No	No	Normal	Surgery+AMB 1500 mg	Response	2 years
10/ Fialk et al., 1981	18/M	Left tibia	65	No	No	Normal	Surgery+AMB 1500 mg	Response	2 years
11/ Galloway and Schochet, 1981	71/M	Right frontal bone	NA	Yes	No	CLL	AMB	Response	NA
12/ Heenan and Dawkins, 1981	54/M	Right os calcis, left tibia, both ulnas	NA	No	Yes	T-cell defect, multiple squamous cell tumors	Surgery	Failure	25 months
							AMB+5-FC 2700 g		
13/ Hammerschlag et al., 1982	11/F	Left femur	40	Yes	No	Normal	AMB 93 mg+5-FC	Response	4 months
14/ Shaff et al., 1982	19/F	Left calcaneus	NA	Yes	No	TB, sarcoidosis, corticosteroid therapy	Surgery+AMB+5-FC	Response	NA
15/ Amenta et al., 1983	33/M	Left femur	Elevated	No	No	Normal	Surgery+AMB	NA	NA
16/ Cash and Goodman, 1983	59/M	Bilateral middle and inner ear	NA	Yes	CNS	Chronic meningitis	AMB+5-FC	Failure	
17/ Perfect et al., 1983	46/F	NA (joint)	NA	NA	CNS, skin, blood	Renal transplantation, corticosteroid therapy	AMB+5-FC	Failure	
18/ Perfect et al., 1983	32/F	Polyarthritis	NA	NA	Retina, CNS, urine, blood	SLE, corticosteroid therapy	AMB	Failure	6 weeks
19/ Perfect et al., 1983	47/M	Knee, ankle, wrist	NA	NA	Blood	Renal transplantation, corticosteroid therapy	AMB+5-FC	Response	4 years
20/ Bunning and Barth, 1984	54/M	Left knee	NA	No	Skin	DM, HTN, cardiomyopathy	Surgery	Response	22 months
							AMB+5-FC		
21/ Reinig et al., 1984	10/F	Left parietal bone	NA	Yes	No	SLE, corticosteroid therapy	Surgery	Failure	
							AMB+5-FC		
22/ Matsushita and Suzuki, 1985	50/M	T9, T10, T11	30	Yes	No	DM, hepatitis, pulmonary silicosis	Surgery×2, 5-FC 2202 g+AMB 1105 mg	Response	21 months
23/ Levine et al., 1985	35/F	Left humerus	30	Yes	No	Sarcoidosis	Surgery+AMB	Response	NA
24/ Brand et al., 1985	26/F	Left sacroiliac joint, left ilium	31	Yes	Yes	Haemolytic anemia, corticosteroid therapy	Surgery+AMB+5-FC	Response	NA
25/ Gould and Gould, 1985	NA	Orbit	NA	NA	NA	NA	AMB+ketoconazole	NA	NA
26/ Zach and Penn, 1986	13/F	Right femur	50	Yes	No	Normal	AMB 1321 mg+5-FC	Response	2 years
27/ Ricciardi et al., 1986	37/M	Right knee	NA	NA	CNS, skin, blood	AIDS, IV drug abuse	AMB+5-FC	Failure	
28/ Baldwin et al., 1988	10/F	Right ilium	111	Yes	No	Normal	Surgery	Response	NA
							AMB 504 mg+5-FC 224 g		
29/ Govender et al., 1988	5/F	Left femur	51	Yes	No	Normal	Surgery	Response	18 months
30/ Govender et al., 1988	29/F	Right ilium	60	No	No	Normal	Surgery	Response	18 months
							AMB+5-FC		
31/ Stead et al., 1988	56/F	Left humerus and shoulder joint; right ischium and hip joint	NA	Yes	Yes	Normal	Surgery+ketoconazole	Response	1 years
32/ Stead et al., 1988	4/M	Left elbow, right knee, right elbow	NA	Yes	Skin	TB, kwashiorkor, chronic otitis	Surgery	Response	NA
							AMB+ketoconazole		
33/ Sinnott and Holt, 1989	54/F	Right knee, metacarpophalangeal joint	NA	No	skin	Renal transplantation, acute gout, corticosteroid therapy	AMB+5-FC	Response	6 months
34/ Lie et al., 1989	27/F	L2, L3, L4, L5	Normal	No	No	Normal	AMB+5-FC	Response	2 months
35/ Behrman et al., 1990	47/M	Right knee	NA	Yes	No	TB	Surgery+AMB	Response	6 months
36/ Kromminga et al., 1990	84/M	Rib, T10, T11, sacrum, femur	NA	Yes	Yes	DM, lung cancer	No	Failure	
37/ Pirofski and Casadevall, 1990	45/M	L1, L2, L3	NA	Yes	CNS	AIDS, IV drug abuse, staphylococcal epidural abscess	Surgery+AMB	Response	1 years
38/ Dounis et al., 1991	55/F	Skull, patella, femur	NA	No	CNS	Normal	Surgery+AMB +5-FC	Response	7 years
39/ Abdul-Karim et al., 1991	9/M	Left scapula	Elevated	Yes	No	IL-2 deficiency	Surgery+AMB	Response	NA
40/ Ueda et al., 1992	58/M	Right tibia	41	Yes	No	Normal	Surgery×2+ketoconazole	Response	2 years
41/ Sorensen et al., 1992	10/M	Left scapula	NA	Yes	No	IL-2 deficiency	AMB+5-FC	Response	4 years
42/ Magid and Smith, 1992	54/F	Left clavicle	NA	Yes	No	DM	AMB+5-FC	Response	10 months
43/ Armonda et al., 1993	39/M	Left temporal bone	Elevated	Yes	Skin	Normal	Surgery+AMB+5-FC	Response	9 months
44/ Gurevitz et al., 1994	67/F	L3	70	Yes	Lung	Normal	AMB 1000 mg+5-FC	Response	2 years
45/ Bosch et al., 1994	55/F	Right ischium, right hip	NA	Yes	CNS	DM	Surgery	Response	7 years
							AMB+5-FC+ketoconazole		
46/ Glynn et al., 1994	52/F	L1, L2	NA	No	CNS	Normal	AMB+5-FC+ketoconazole	Response	7 years
47/ Singh et al., 1994	56/M	Ankle	NA	NA	Skin, lung, blood	Liver transplantation, corticosteroid therapy	AMB+Flu+5-FC+ itraconazole	Response	6 months
48/ Schmidt et al., 1995	53/F	Left femur, skull, left humerus, C5, C6	NA	No	Yes	Normal	AMB	Response	NA
							1095 mg+5-FC+Flu		
							Surgery		
49/ Wood and Miedzinski, 1996	49/M	Left temporal	NA	No	No	Lymphopenia, hepatitis	Surgery	Response	18 months
							AMB 300 mg+Flu		
50/ Hummel et al., 1996	43/M	Left femur	NA	Yes	No	Sarcoidosis, corticosteroid therapy	Flu	Response	NA
51/ Benard et al., 1996	57/M	Frontal bone, mandible	NA	No	No	Corticosteroid therapy	AMB+itraconazole	Response	2 years
52/ Kumlin et al., 1997	79/M	Right knee	105	Yes	No	Lymphopenia	AMB+5-FC	Response	2 years
53/ Mauri et al., 1997	41/M	Knee	NA	NA	CNS	AIDS	AMB 1500 mg+Flu	Response	12 months
54/ Liu, 1998	60/M	Right humerus, right tibia	NA	Yes	Yes	TB, lymphadenitis	Surgery+AMB	Failure	3 months
55/ Raftopoulos et al., 1998	14/F	10th left rib	22	Yes	No	Normal	Surgery+AMB+Flu	Response	7 months
56/ Case Records of the Massachusetts General Hospital, 1999	55/M	Right tibia	NA	Yes	Lung, skin	DM, renal transplantation, corticosteroid therapy	Surgery+Flu	Response	NA
57/ Jain et al., 1999	72/F	T6	70	No	lung	DM, TB	AMB+5-FC	Response	5 years
58/ Noh et al., 1999	21/F	Left sacrum	NA	No	CNS	Hepatitis, corticosteroid therapy	Surgery×2+AMB	Response	3 years
59/ Witte et al., 2000	68/M	Left humerus	NA	Yes	No	DM	NA	NA	NA
60/ Prendiville et al., 2000	48/F	Sphenoid sinus, skull base	NA	Yes	CNS	Sinusitis, septicemia, corticosteroid therapy	Surgery×2 Flu+AMB	Response	NA
61/ Cook, 2001	24/F	T1, T2, T3	NA	Yes	No	Sarcoidosis	Surgery+Flu+5-FC+AMB	Response	16 months
62/ Italiano et al., 2001	37/F	Left knee	32	NA	No	Sarcoidosis, Sjogren's syndrome, corticosteroid therapy	AMB+5-FC	Response	NA
63/ Zanelli et al., 2001	27/F	Left ilium, left acetabulum	Elevated	Yes	Muscles	Lymphopenia	Surgery	Response	1 years
							AMB3 g+Flu+itraconazole		
64/ Bruno et al., 2002	42/M	Left elbow joint, left wrist	NA	No	tendon	DM, renal transplantation, corticosteroid therapy	Surgery+Flu	Response	6 months
65/ Gupta et al., 2003	24/F	T1, T2, T3, 3rd rib	NA	NA	Yes	TB	Surgery+AMB+5-FC	Failure	2 weeks
66/ Ching et al., 2004	17/F	Right posterior parietal	46	Yes	CNS	AML, corticosteroid therapy	Surgery+AMB+5-FC+Flu	Response	NA
67/ Wildstein et al., 2005	20/M	T12, L1, L2	36	Yes	No	Sarcoidosis, corticosteroid therapy	Flu	Response	6 months
68/ Chang et al., 2005	22/M	Left 9th rib	19	Yes	Pleural	Normal	Surgery+AMB20 mg+Flu	Response	12 months
69/ Goldshteyn et al., 2006	19/F	Left humeral head	28	Yes	Urine	Sarcoidosis, corticosteroid therapy	AMB+Flu	Response	1 months
70/ Hawkins and Flaherty, 2007	84/F	Left 3rd digit	NA	Yes	CNS	BP, CHF, DJD, DM, hypothyroidism, corticosteroid therapy	Surgery×2 AMB+5-FC+Flu	Failure	2 months
71/ Al-Tawfiq and Ghandour, 2007	34/F	L4, L5	89	Yes	No	TB	Surgery+Flu	Response	12 months
72/ Amit et al., 2008	38/F	Frontoparietal joint	NA	Yes	No	Lymphopenia	Surgery+Flu	Response	NA
73/ Saeed et al., 2009	54/F	Right frontal bone	NA	No	No	HTN	Surgery+AMB+5-FC+Flu	Response	6 weeks
74/ Burton et al., 2009	35/M	Sternal notch, left elbow, left 5th and 6th ribs	NA	No	Skin, CNS	TB, AIDS	Surgery+AMB+Flu	Response	19 months
75/ Geller et al., 2009	38/M	Left clavicle, sternoclavicular joint	25	No	Yes	Testicular cancer, sarcoidosis	Surgery+Flu	Response	NA
76/ Agadi et al., 2010	42/M	Right frontal bone	NA	Yes	CNS	DM, TB, lymphopenia, renal carcinoma	Flu	Response	NA
77/ Singh and Xess, 2010	29/F	L5, sternum	NA	No	Yes	TB, pregnancy	AMB	Failure	
78/ Houda et al., 2011	70/F	T8, T9, T10	100	No	CNS	Normal	Surgery+AMB+Flu	Response	NA
79/ McGuire et al., 2011	10/F	Left iliac crest	99	No	No	Normal	Surgery×2+Flu	Response	7 months
80/ Jain et al., 2011	43/F	Proximal phalanx of middle finger	35	Yes	No	Normal	Surgery+AMB+Flu	Response	6 months
81/ Qadir et al., 2011	28/F	Left distal radius	26	Yes	No	Normal	Flu	Response	12 weeks
82/ Corral et al., 2011^*^	65/M	Right parietal bone	NA	Yes	No	Normal	Surgery+AMB+Flu	Response	2 months
83/ Jou et al., 2011	50/M	Right femur, right 7th rib	NA	Yes	Yes	Normal	Surgery+Flu	Response	5 years
84/ Zhang et al., 2012	57/F	Left scapula, left 6th rib	76	Yes	Yes	HTN	AMB+5-FC+Flu	Response	2 years
85/ Jacobson et al., 2012	27/M	Right femur	59	Yes	No	Normal	Surgery+Flu	Response	8 months
86/ Flannery et al., 2012	65/F	T2	34	Yes	No	DM	AMB+5-FC+Flu	Response	NA
87/ Ramkillawan et al., 2013	56/M	Left humerus	NA	Yes	No	Normal	Surgery+AMB+Flu	Response	NA
88/ Jain et al., 2013	41/F	Sternum	30	Yes	No	Normal	Surgery+Flu+AMB+5-FC	Response	1 years
89/ Zhou et al., 2013	40/F	L4	22	No	No	Rheumatoid arthritis, scleroderma	Flu	Response	12 months

*NA, not available; 5-FC, 5-fluorocytosine; CLL, chronic lymphocytic leukemia; TB, tuberculosis; SLE, systemic lupus erythematosus; DM, diabetes mellitus; HTN, hypertension; IL-2, interleukin-2; Flu, fluconazole; AML, acute myeloid leukemia; BP, bullous pemphigoid; CHF, congestive heart failure; DJD, degenerative joint disease*.

* *Variety identification taken*.

### Sex and age

**Figure 1:**
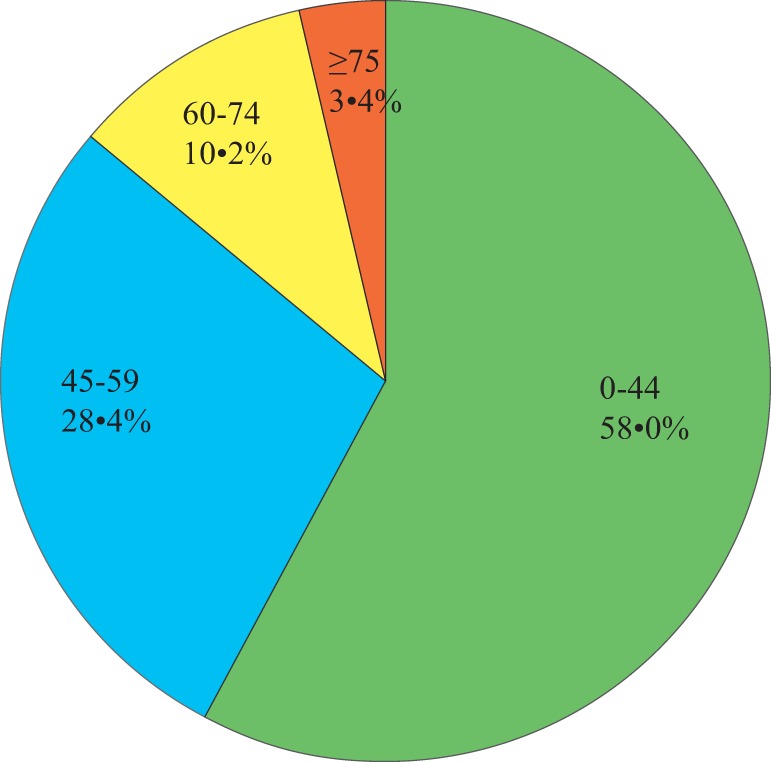
**Pie chart of the 88 patients' age**.

Forty-four (of 88, 50.0%) males and 44 (of 88, 50.0%) females were included. Their ages ranged from 16 months to 84 years with a mean (±SD) of 39.9 years (±19.6; Figure 1). Relatively immunocompromised hosts (*n* = 31, 48.8 ± 17.9 years) were older than immunocompetent hosts (*n* = 32, 33.9 ± 19.7 years; ANOVA, *p* = 0.002) and classically immunodeficient hosts (*n* = 25, 36.7 ± 18.5 years; ANOVA, *p* = 0.018); however, classically immunodeficient hosts were approximately the same age as the immunocompetent hosts (ANOVA, *p* = 0.587).

### Epidemiological histories

Thirteen (of 88, 14.8%) patients had epidemiological histories (Table 2). The epidemiological histories of the patients with immune abnormalities (seven of 56, 12.5%) and those who were immunocompetent (six of 32, 18.8%) did not significantly differ (χ^2^-test, *p* = 0.629).

**Table 2:** **Epidemiological histories of 13 patients**.

**Case no./References**	**Age/Sex**	**Epidemiological histories**	**Immune status**
2/ Chleboun and Nade, 1977	40/F	Contact with soil (farmer)	Sarcoidosis
52/ Kumlin et al., 1997	79/M	Contact with soil (farmer)	Lymphopenia
56/ Case Records of the Massachusetts General Hospital, 1999	55/M	Contact with soil (farmer)	Renal transplantation
82/ Corral et al., 2011	65/M	Contact with soil (agricultural worker), chronic trauma (1 year)	Immunocompetent
87/ Ramkillawan et al., 2013	56/M	Contact with soil (agricultural worker)	Immunocompetent
38/ Dounis et al., 1991	55/F	Chronic trauma	Immunocompetent
62/ Italiano et al., 2001	37/F	Acute trauma	Sjogren's syndrome
68/ Wildstein et al., 2005	22/M	Chronic trauma (4 weeks)	Immunocompetent
26/ Zach and Penn, 1986	13/F	Exposure to sea gull, chickens	Immunocompetent
39/ Abdul-Karim et al., 1991	9/M	Exposure to sparrows droppings	Interleukin-2 deficiency
49/ Singh and Xess, 2010	49/M	Exposure to pigeons	Lymphopenia
60/ Prendiville et al., 2000	48/F	Exposure to a parakeet	Chronic sinusitis
85/ Jacobson et al., 2012	27/M	Exposure to bird droppings	Immunocompetent

### Involved sites

**Figure 2:**
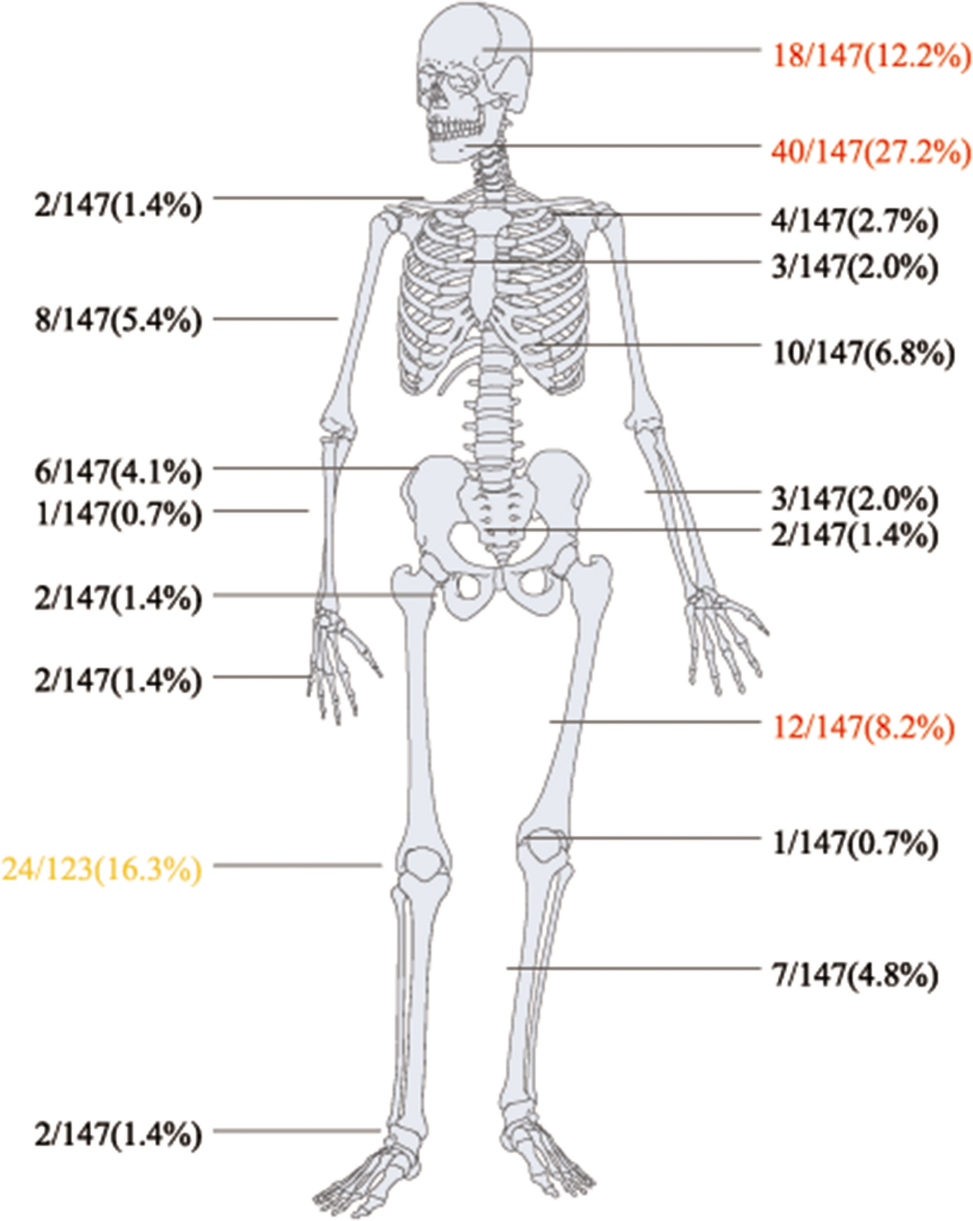
**Bones and joints involved (147 sites) in skeletal cryptococcal lesions**.

**Figure 3:**
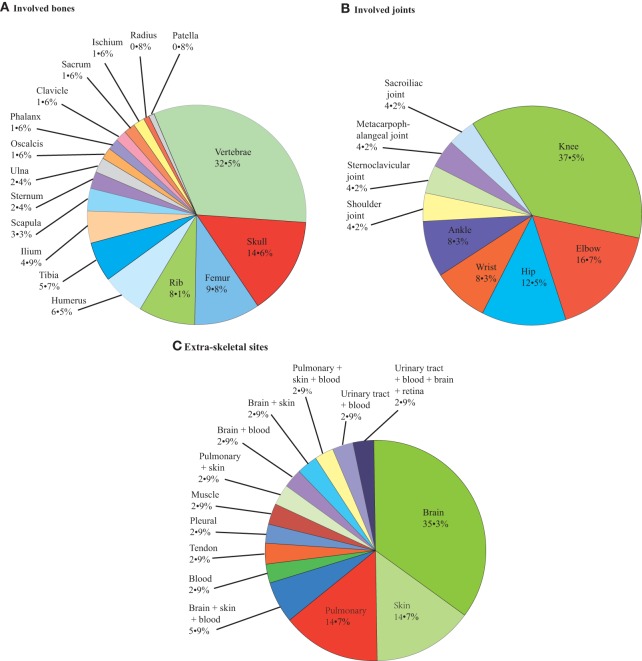
**Pie chart of patients' involved bones **(A)**, joints **(B)** and extra-skeletal sites **(C)**: 123, 24, 34, respectively**.

Regarding bone and joint infections, see Figures 2, 3A,B. Multiple site infections did not differ among the classically immunodeficient (10 of 25, 40.0%), relatively immunocompromised (13 of 29, 44.8%), and immunocompetent groups (10 of 32, 31.3%; χ^2^-test, *p* = 0.542). Extra-skeletal cryptococcosis was found in 34 patients (Figure 3C). The patients categorized as classically immunodeficient (16 of 25, 64.0%) were more likely to have extra-skeletal infections than were those categorized as relatively immunocompromised (10 of 31, 32.3%; χ^2^-test, *p* = 0.018) and immunocompetent (eight of 32, 25.0%; χ^2^-test, *p* = 0.003), whereas patients categorized as relatively immunocompromised or immunocompetent did not differ in this regard (χ^2^-test; *p* = 0.524). Excluding case 25, 42 (of 88, 47.7%) patients had disseminated cryptococcosis. The patients categorized as classically immunodeficient (17 of 25, 68.0%) were more likely to have disseminated cryptococcosis than were those categorized as immunocompetent (11 of 32, 33.4%; χ^2^-test; *p* = 0.012). Dissemination among the patients categorized as classically immunodeficient did not differ from that among those categorized as relatively immunocompromised (14 of 31, 45.2%; χ^2^-test; *p* = 0.087), nor did the dissemination among the patients categorized as relatively immunocompromised differ from that among those categorized as immunocompetent (χ^2^-test; *p* = 0.382).

### Clinical symptoms

The predominant complaints included soft tissue swelling and pain, the duration of which ranged from acute admission to 3 years. Of the evaluable 86 patients (excluding cases 25, 41, and 50 whose data were not available), fever was observed in 18 (20.9%) patients, but body temperature measurements were only available in 12 of these patients (66.7%) and ranged from 37.4°C to 39.2°C (mean temperature = 38.35 ± 0.61°C).

### Underlying diseases

Of the 88 patients (excluding case 25), 25 (28.4%) were categorized as classically immunodeficient, 31 (35.2%) were relatively immunocompromised, and 32 (36.7%) were immunocompetent. Corticosteroid use (18 of 25, 72.0%) was the most common cause of classically immunodeficient status. Diabetes mellitus (nine of 31, 29.0%) followed by tuberculosis (seven of 31, 22.6%) and connective tissue disorders (five of 31, 16.1%) were the most common causes of the relatively immunocompromised status. Of the 32 patients in the immunocompetent group, 22 (68.8%) were described in articles published before 1999, and 10 (31.2%) were described in articles published after 2004. No immunocompetent patients were described between 1999 and 2004.

### Laboratory tests

The erythrocyte sedimentation rate (ESR) was documented for 40 (of 89, 44.9%) patients. Thirty-seven (of 40, 92.5%) ESRs were elevated (i.e., >20 mm/h for females and >15 mm/h for males) for 24 (of 37, 64.9%) female patients and 13 (of 37, 35.1%) male patients. Among the 24 female patients, definite elevated ESRs were documented in 23 (of 24, 95.8%) patients (average, 52.0 mm/h ± 27.3), whereas among the 13 male patients, definite elevated ESRs were documented in 10 (of 13, 76.9%) patients (average, 49.0 mm/h ± 30.5).

The diagnostic specimens were most often obtained from open biopsies, followed by aspiration and incision and drainage (Table 3). All 67 cases with fungal cultures showed positive results. Of the 53 histopathological analyses, the diagnostic specimens were obtained from open biopsies in 37 (69.8%) cases, aspiration in 13 (24.5%) cases, and incision and drainage in three (5.7%) cases; positive results were obtained for 21 (of 37, 56.8%) cases, seven (of 13, 53.8%) cases, and one (of three, 33.3%) case, respectively. Positive histopathological analyses showed foreign-body giant cells and capsulated yeast-like organisms. The capsule structure was stained using periodic acid Schiff (PAS) stain in 22 (of 29, 75.9%) patients, mucicarmine stain in 13 (of 29, 44.8%) patients, Gomori's Methenamine silver (GMS) stain in 18 (of 29, 62.1%) patients, Masson-Fontana silver stain in case 15, and colloidal iron techniques in case 23.

**Table 3:** **Diagnostic modalities in 82 patients with skeletal cryptococcosis**.

**Modality**	**No. of tests**	**Culture **n** (%)**	**Histopathological analysis **n** (%)**	**Both **n** (%)**
Open biopsy	50	13 (26.0)	10 (20.0)	27 (54.0)
Aspiration	27	14 (51.9)	5 (18.5)	8 (29.6)
Incision and drainage	5	2 (40.0)	0 (0)	3 (60/0)
Total No.	82	29 (35.4)	15 (18.3)	38 (46.3)

Only two patients with immunocompetent status (cases 44 and 82) had their strains successfully identified, using cultures on dihydroxyphenylalanine (DOPA) and canavanine glycine bromothymol (CGB) blue agars; both patients were infected with CNVN.

### Radiological manifestations

Of the 89 patients, 77 (86.5%) had one or more radiological examinations of their affected bones. Sclerosis was observed in the relatively immunocompromised cases 23 and 45; periosteal reaction was described in 13 (of 76, 17.1%) patients. Subperiosteal new bone formation was noted in case 79 (immunocompetent), and irregular cortical destruction and extensive periosteal reaction was noted in case 59 (relatively immunocompromised).

Of the 80 evaluable patients, osteomyelitis was found in 51 (63.8%) patients. Case 41 was documented as having osteomyelitis, and the other 50 patients were diagnosed based on either a positive radiograph or bone scan; five (of 50, 10.0%) patients were also diagnosed based on a positive histopathology. The presence of osteomyelitis among patients categorized as classically immunodeficient (11 of 18, 61.1%), relatively immunocompromised (20 of 30, 66.7%), and immunocompetent (20 of 32, 62.5%) did not differ (χ^2^-test; *p* = 0.911).

### Delays in diagnosis

Of the 88 evaluable patients (excluding case 36, that was diagnosed post-mortem), delays in diagnosis occurred among 20 (of 88, 22.7%) patients. The delayed time of these 20 patients (documented in only 14 patients) ranged from 6 days to 10 months. In addition, 13 (of 20, 65.0%) patients were initially misdiagnosed (Table 4), most commonly with tuberculosis (6 of 13, 46.2%) primarily occurring in the vertebrae (5 of 6, 83.4%). The location of the source of discomfort was not reported for the remaining 7 (of 20, 35.0%) patients.

**Table 4:** **Misdiagnosis of 11 patients**.

**Case no./References**	**Bone and joints**	**Misdiagnosis**	**Delay time**	**Treatment before diagnosis**	**Method of definite diagnosis**	**Treatment after diagnosis**	**Outcome**
22/Matsushita and Suzuki, 1985	T9, T10, T11	Metastatic cancer	7 months	Irradiation ATT, immobilization	Histopathology	Surgery, 5-FC+AMB	Response
		Tuberculosis					
28/Baldwin et al., 1988	Right ilium	Musculoskeletal pain	6 weeks	Acetaminophen	Histopathology culture	Surgery, AMB504 mg+5-FC224 g	Response
29/Govender et al., 1988	Left femur	Bacterial osteomyelitis	NA	Antibiotics immobilization	Culture	Surgery	Response
32/Stead et al., 1988	Left elbow, right knee and right elbow	Tuberculosis	18 weeks	ATT, antibiotics, physiotherapy	Culture	Surgery AMB+ketoconazole	Response
48/Schmidt et al., 1995	Left femur, skull, left humerus, C5, C6	Tuberculosis	16 days	ATT	Culture	AMB+5-FC+Flu	Response
54/Liu, 1998	Right humerus right tibia	Bacterial infection	>1 months	Incision and drainage	Histopathology culture	Surgery+AMB	Failure
57/Jain et al., 1999	T6	Tuberculosis	>3 months	ATT	Histopathology culture	AMB+5-FC	Response
60/Prendiville et al., 2000	Sphenoid sinus, skull base	Tolosa-Hunt syndrome	>2 months	Prednisone	Histopathology culture	Surgery+Flu+AMB	Response
65/Gupta et al., 2003	T1, T2, T3	Tuberculosis	NA	ATT	Histopathology	Surgery	Failure
69/Goldshteyn et al., 2006	Left humeral head	An avascular necrosis		NSAIDs	Culture	AMB+Flu	Response
77/Agadi et al., 2010	L5, sternum	Tuberculosis	NA	ATT	Histopathology culture	AMB	Failure
82/Corral et al., 2011	Right parietal bone	Soft tissue infection	>10 months	Antibiotics	Histopathology culture	Surgery+AMB+Flu	Response
88/Jain et al., 2013	Sternal	Gastroesophageal reflux disease	NA	Antacids	Histopathology culture	Surgery+Flu+AMB+5-FC	Response

*ATT, antituberculosis therapy; 5-FC, 5-fluorocytosine; AMB, amphotericin B; NA, not available; Flu, fluconazole; NSAIDs, nonsteroidal anti-inflammatory medications*.

### Treatment, follow-up assessment, and outcomes

Of the evaluable 87 patients (excluding patient 36 who did not receive any treatment and was diagnosed at autopsy as well as patient 59 whose treatment information was not available), 80 (of 87, 92.0%) patients received one treatment regimen and the other 7 (of 87, 8.0%) patients changed treatments because their symptoms became aggravated or recurred.

Of the 80 patients who received only one treatment regimen, 3 (of 80, 3.8%) patients received surgery alone, 32 (of 80, 40.0%) patients received medical treatment alone and 45 (of 80, 56.2%) patients received surgery in conjunction with medical treatment. Of the 3 patients that underwent surgery alone, 1 (of 3, 33.3%) patient died and 2 (of 3, 66.7%) patients responded. Of the 32 patients treated with medical treatment alone, 10 patients (31.3%) received monotherapy (AMB was the most commonly used treatment; 5 of 10, 50.0%) and three patients (30.0%) died. Twenty-two (of 32, 68.7%) patients received combined therapies (AMB plus 5-FC was the most commonly used treatment; 14 of 22, 36.4%), three of whom died (13.6%). Of the 45 patients treated with surgery and medical treatments, 18 patients (40.0%) underwent surgery and monotherapy (AMB was most commonly used; 10 of 18, 55.6%), one of whom died (5.6%). A total of 27 (of 45, 60.0%) patients underwent surgery combined with several medicines (AMB plus 5-FU was the most commonly used treatment; 11 of 27, 40.7%), four of whom died (14.8%).

In total, 68 patients responded to treatment, and 12 patients died (Table 5). Improvements of the symptoms and the rclinical signs of all 68 patients who responded were observed. Only six of these patients' (8.8%) ESRs were measured after treatment, all of which were normal or decreased. Only 13 (of 68, 19.1%) patients underwent serum cryptococcal antigen testing, and all of these patients showed reductions or undetectable levels. X-rays, CT, or MRI scans were obtained in 21 (of 68, 30.9%) patients, and all of them showed healing or resolution. Bone scans were performed in two (of 68, 2.9%) patients, and both of them presented reduced isotope uptake. Six of the 12 patients who received treatment but died were classically immunodeficient (50.0%), five were relatively immunocompromised (41.7%), and case 4 was immunocompetent (Table 6). In addition, nine (of 12, 75.0%) patients presented with disseminated cryptococcosis. Only one of 12 patients' deaths was directly caused by cryptococcosis (8.3%; case 65, relatively immunocompromised).

**Table 5:** **Treatment of the 80 patients**.

**Treatment**	**Definite medical therapy**	**No. of patients**	**Outcome**	
			**Response n (%)**	**Failure n (%)**
Surgery (*n* = 3)		3	2 (66.7)	1 (33.3)
Medical treatment (*n* = 32)	AMB	5	2 (40.0)	3 (60.0)
	AMB+5-FC	14	11 (78.6)	3 (21.4)
	Flu	5	5 (100.0)	0 (0)
	AMB+Flu	2	2 (100.0)	0 (0)
	AMB+itraconazole	1	1 (100.0)	0 (0)
	AMB+5-FC+Flu	2	2 (100.0)	0 (0)
	AMB+ketoconazole	1	1 (100.0)	0 (0)
	AMB+5-FC+ketoconazole	1	1 (100.0)	0 (0)
	AMB+5-FC+Flu+itraconazole	1	1 (100.0)	0 (0)
		32	26 (81.2)	6 (18.8)
Surgery and medical treatment (*n* = 45)	AMB	10	9 (90.0)	1 (10.0)
	AMB+5-FC	11	8 (72.7)	3 (27.3)
	Flu	6	6 (100.0)	0 (0)
	AMB+Flu	8	8 (100.0)	0 (0)
	AMB+5-FC+Flu	5	4 (80.0)	1 (20.0)
	Ketoconazole	2	2 (100.0)	0 (0)
	AMB+ketoconazole	1	1 (100.0)	0 (0)
	AMB+5-FC+ketoconazole	1	1 (100.0)	0 (0)
	AMB+Flu+itraconazole	1	1 (100.0)	0 (0)
		45	40 (88.9)	5 (11.1)
Total No.		80	68 (85.0)	12 (15.0)

*AMB, amphotericin B; 5-FC, 5-fluorocytosine; Flu, fluconazole*.

**Table 6:** **Twelve deceased patients**.

**Case no./References**	**Age/Sex**	**Disseminated or not**	**Immune status**	**Treatment**	**Cause of death**
1/ Chleboun and Nade, 1977	43/M	No	Relatively immunocompromised	Surgery	Unknown
4/ Chleboun and Nade, 1977	15/M	No	Immunocompetent	AMB395 mg	Tuberculous hepatitis and staphylococcal pneumonia
12/ Heenan and Dawkins, 1981	54/M	Yes	Classically immunodeficient	Surgery AMB+5-FC2700 g	Unknown
16/ Cash and Goodman, 1983	59/M	CNS	Relatively immunocompromised	AMB+5-FC	Cardiorespiratory arrest on the 13th day of therapy
17/ Perfect et al., 1983	46/F	CNS, skin, blood	Classically immunodeficient	AMB+5-FC	Serratia septicemia
18/ Perfect et al., 1983	32/F	Retina, CNS, urine, blood	Classically immunodeficient	AMB	Unknown
21/ Reinig et al., 1984	10/F	No	Classically immunodeficient	Surgery AMB+5-FC	Respiratory failure
27/ Ricciardi et al., 1986	37/M	CNS, skin, blood	Classically immunodeficient	AMB+5-FC	Unknown
54/ Liu, 1998	60/M	Yes	Relatively immunocompromised	Surgery AMB	Severe hepatic failure
65/ Gupta et al., 2003	24/F	Yes	Relatively immunocompromised	Surgery AMB+5-FC	Cryptococcosis
70/ Goldshteyn et al., 2006	84/F	CNS	Classically immunodeficient	Surgery×2 AMB+5-FC+Flu	Unknown
77/ Agadi et al., 2010	29/F	Yes	Relatively immunocompromised	AMB	Cardiac failure

*AMB, amphotericin B; 5-FC, 5-fluorocytosine; Flu, fluconazole*.

Of the seven patients who changed treatment, symptom aggravation during primary treatment occurred among five (of seven, 71.4%) patients, one of whom (case 22, relatively immunocompromised) underwent an ESR examination that revealed an ESR increase from 30 mm/h to >80 mm/h after the administration of 917 mg of AMB for 14 weeks. Symptoms recurred for case 7 (immunocompetent) and case 60 (relatively immunocompromised) after primary treatment, but neither met the criteria for relapse. Of these seven patients, one case (case 58) was categorized as classically immunodeficient, two were categorized as relatively immunocompromised, and the other four (57.1%) were categorized as immunocompetent. Three (of seven, 42.9%) patients presented with dissemination. All seven patients responded well to the subsequent treatment.

The follow-up time ranged between 2 weeks and 15 years (median = 13.5 months); half of all patients (30 of 60) were followed-up for less than 1 year, and the other half were followed-up for more than 1 year.

The factors associated with the overall skeletal cryptococcosis mortality rate, stratified by response to treatment, are listed in Table 7. Dissemination was a risk factor the overall mortality rate (*p* = 0.041); the patient immune status was not a risk factor mortality (*p* = 0.056).

**Table 7:** **Factors associated with overall skeletal cryptococcosis mortality (excluding cases 25, 36, and 59 and the 12 deceased patients)**.

**Characteristics**	**Non-survival (%)**	**Survival (%)**	***P*-value**
N	67	12	
Mean age	39.2 ± 19.1	41.1 ± 21.1	0.752
No. Male	33 (49.3%)	5 (41.7%)	0.628
Epidemiological histories	11 (16.4%)	0 (0)	0.289
Multiple site infections	23 (34.3%)	6 (50.0%)	0.476
Extra-skeletal infections	25 (37.3%)	6 (50.0%)	0.612
Dissemination	28 (41.8%)	9 (75.0%)	0.034
Immune abnormality	40 (59.7%)	11 (91.7%)	0.071
Delay in diagnosis	14 (20.9%)	3 (25.0%)	1.000

Finally, to compare the mortality rates associated with different treatments, we analyzed 40 patients who were treated with AMB alone (2 of 5, 40.0%), AMB plus 5-FC (11 of 14, 78.6%), surgery combined with AMB (9 of 10, 90.0%), or surgery combined with AMB plus 5-FC (8 of 11, 72.7%); these cases were chosen because these three treatments (AMB, 5-FC and surgery) were utilized more often than other therapies. Specific information is listed in Table 8. The mortality rates of the four treatment regimens did not differ (*p* = 0.229), and dissemination predicted mortality (*p* = 0.044).

**Table 8:** **Factors associated with 40 patients treated with AMB alone, AMB plus 5-FU, surgery combined with AMB, or surgery combined with AMB plus 5-FU**.

**Characteristics**	**Non-survival (%)**	**Survival (%)**	***P*-value**
N	30	10	
Mean age	36.2 ± 22.4	36.6 ± 17.8	0.964
No. Male	16 (53.3%)	4 (40.0%)	0.465
Epidemiological histories	5 (16.7%)	0 (0)	0.408
Multiple site infections	8 (26.7%)	6 (60.0%)	0.126
Extra-skeletal infections	11 (36.7%)	5 (50.0%)	0.709
Dissemination	11 (36.7%)	8 (80.0%)	0.044
Immune abnormality	16 (53.3%)	9 (90.0%)	0.090
Delay in diagnosis	7 (23.3%)	3 (30.0%)	1.000
Treatment AMB	2 (6.7%)	3 (30.0%)	0.229
AMB+5-FC	11 (36.7%)	3 (30.0%)	
surgery+AMB	9 (30.0%)	1 (10.0%)	
surgery+AMB+5-FC	8 (26.7%)	3 (30.0%)	

## Discussion

Because knowledge regarding the clinical features, treatment, and prognosis of skeletal cryptococcosis is limited, this large-scale systematic analysis of previously reported skeletal cryptococcosis was conducted to better understand the disease.

Skeletal cryptococcosis affects both individuals who are immunocompetent and those with abnormal immunity (Behrman et al., 1990; Zhang et al., 2012). Our study revealed immune status to be an important risk factor for this infection, which is consistent with previous reports (Hawkins and Armstrong, 1984; Jacobson et al., 2012). Most of the patients with immune abnormalities included in this study had defects of cellular immunity such as those related to lymphoma, leukemia, sarcoidosis, and long-term steroid use. Cellular immunity defects might predispose patients to cryptococcal infection, which can lead to T cell abnormalities in hosts without other underlying diseases. This possibility suggests that T cell mediated immunity is the primary pathway for preventing cryptococcal infections (Meredith et al., 1979; Agadi et al., 2010; Jacobson et al., 2012). Thus, once a patient is suspected with cryptococcal infection, the evaluation of lymphocyte subsets, including counts and stimulation studies, should be routinely performed to specifically and sensitively reveal the patient immune status, as suggested by Wood and Miedzinski (1996).

Since the introduction of highly active antiretroviral therapy (HAART) in 1995, the mortality rate associated with AIDS has dramatically decreased (Mitchell and Perfect, 1995). Subsequently, steroids are considered the leading cause of skeletal cryptococcosis because of their extensive use for both therapeutic and recreational purposes (Benard et al., 1996; Hummel et al., 1996). In addition, the incidence of chronic diseases such as diabetes mellitus and hypertension has increased (Jain et al., 1999; Witte et al., 2000; Bruno et al., 2002). The number of patients with cryptococcal disease and who are classified as immunocompetent has risen greatly since 2004 and is estimated to increase by 0.2 per million every year (Zhang et al., 2012; Jain et al., 2013; Zhou et al., 2013). This increase was most likely due to the CNVG outbreak that originated in 1999 and resurged in 2004 (Chaturvedi and Chaturvedi, 2011). However, determining the reasons why patients with immunocompetent statuses were not found between 1999 and 2004 is difficult. CNVN, which is ecologically widespread and exists in soil contaminated by pigeon excreta, is more common in immunocompromised patients with cell-mediated immune deficiencies, whereas CNVG, which is traditionally found in eucalyptus trees located in tropical and subtropical areas (Speed and Dunt, 1995; Chaturvedi and Chaturvedi, 2011; Harris et al., 2011) (the south-eastern region of China is the most common location (Chen et al., 2008; Negroni, 2012), affects immunocompetent hosts. Thus, it is essential for clinical microbiology laboratories to accurately differentiate CNVG from other forms of *C neoformans* to determine the final diagnosis and guide the initiation of or institute the appropriate treatment (Klein et al., 2009; Singh and Xess, 2010). Recently, a testing survey conducted by the New York State Department of Health indicated that only 5.0% of clinical laboratories were able to correctly identify CNVG (Klein et al., 2009). CNVN and CNVG are commonly differentiated by DOPA agar and CGB agar, where different colored reactions can be observed (Klein et al., 2009; Qadir et al., 2011). The results are often available within 48 h; CNVG produces a blue color, whereas CNVN fails to cause a color change. Furthermore, Klein and colleagues first used the specific method of D2 large ribosomal subunit region sequencing to identify CNVG (Klein et al., 2009). McTaggart and colleagues explored a cost-effective method called matrix-assisted laser desorption/ionization time-of-flight mass spectrometry (MALDI-TOF MS) (McTaggart et al., 2011). Feng X provided a rapid, simple, and reliable method using Singleplex PCR assay that is suitable for laboratory diagnoses and large-scale epidemiologic studies (Feng et al., 2013). Both strains were shown to have similar susceptibilities to antifungal drugs when tested *in vitro* in some reports (Chen et al., 2000; Thompson et al., 2009). The treatments for patients with disseminated disease due to CNVG are the same as those for CNVN.

Our study did not reveal a relationship between diagnosis and sex, which is consistent with previous reports (Behrman et al., 1990; Wood and Miedzinski, 1996; Zhou et al., 2013). However, Behrman and colleagues reported that 51.0% (20 of 39) patients were males in 1990 (Behrman et al., 1990), whereas Bruno and colleagues reported that 61.9% (13 of 21) patients were males in 2002 (Bruno et al., 2002); these findings might be due to the smaller number of reviewed cases in their studies. Cryptococcal infections occurred in all age groups, with a slight tendency toward for younger people to be affected (e.g., most patients were under 45 years of age). However, the patients with relatively immunocompromised statuses were elderly, which might be explained by the aforementioned leading cause of skeletal cryptococcosis (CNVN) and the fact that older patients are more likely to have chronic diseases.

Skeletal cryptococcosis is usually secondary to hematogenous migration from a primary pulmonary infection after inhaling microscopic, airborne fungal spores (especially after exposure to soil or poultry). These spores are a cause for cryptococcal infection (Armonda et al., 1993; Wood and Miedzinski, 1996). Direct inoculation during trauma is also possible (Chleboun and Nade, 1977; Dounis et al., 1991; Italiano et al., 2001). However, only 14.8% of the patients included in this review had contact with soil or pigeons or a history of trauma; incomplete patient histories might explain this finding. In addition, host immunity did not significantly affect their epidemiological histories. This result differs from that of Jacobson and colleagues, who reported that host immunity effectively excluded infection after initial exposure (Jacobson et al., 2012). This disparity might be due to the recent increase in patients with immunocompetent statuses. Other infectious pathways include direct inoculation through the skin during trauma and contiguous spread combined with the lower involvement of the lymphatic route (Zanelli et al., 2001).

Compared with most of the previous articles that reviewed skull cryptococcosis, articular cryptococcosis, or other bony cryptococcosis alone, such as in Chleboun and Nade's (1977) study, we examined all types of involved sites (Chleboun and Nade, 1977). Any bone or joint can be affected, but the most common site was the vertebrae, which is consistent with Chleboun's report (Chleboun and Nade, 1977); the sufficient blood supply of the vertebrae might explain this finding. The second and third most common sites have changed from the pelvis and rib in 1977 to the skull and femur (Chleboun and Nade, 1977). The most common affected joint was the knee, which is consistent with the study by Bruno et al. (2002). The involvement of multiple bones occurred more regularly in adjacent areas than discrete areas, which indicates the extension of local foci; this result is consistent with Behrman and colleagues' study in 1990 (Behrman et al., 1990). Patients with classically immunodeficient statuses were most likely to have concurrent extra-skeletal involvement sites, and meningitis was the most common extra-skeletal infection. Overall, 47.7% of patients with (especially the classically immunodeficient status) or without immune abnormalities presented with dissemination, and these patients were more likely to show symptom aggravation, recurrence, or death.

The characteristic symptoms of skeletal cryptococcosis are pain and swelling (Chleboun and Nade, 1977; Behrman et al., 1990; Wood and Miedzinski, 1996). Fever, which is not a primary patient complaint (Behrman et al., 1990), was found in only 20.9% of evaluable patients comparable with a previous study, which reported a rate of 18.0% (Wood and Miedzinski, 1996). In addition, classic symptoms such as vomiting (Cash and Goodman, 1983; Agadi et al., 2010), blurred vision (Cash and Goodman, 1983; Prendiville et al., 2000; Ching et al., 2004), dizziness, seizure, diplopia, trismus (Cash and Goodman, 1983), limited motion (Bunning and Barth, 1984; Ricciardi et al., 1986; Sinnott and Holt, 1989), paralysis (Meredith et al., 1979; Gupta et al., 2003), muscle weakness, urinary retention (Gurevitz et al., 1994), and sciatica (Houda et al., 2011) can occur among patients with cryptococcal infections of specific sites; the location can assist in making the final diagnosis.

The ESR can be elevated to various levels when the infection is found in the bone, decline to normal when osseous lesions heal, and increase again when patients have an extensive relapse (Chleboun and Nade, 1977; Behrman et al., 1990; Wood and Miedzinski, 1996).

The diagnosis of skeletal cryptococcosis is primarily based on the examination of lesion specimens from aspiration, surgery, and open biopsies (Behrman et al., 1990; Wood and Miedzinski, 1996; Gupta et al., 2003). Aspiration was the most common method performed in our review, whereas Behrman and colleagues reported that open biopsy was the most commonly performed technique (Behrman et al., 1990); this discrepancy might be attributable to advancements in medical techniques since 1990. All of these methods have a similar diagnostic value. Thus, aspiration is recommended first given its increased convenience and minimal harm caused to the body; however, if aspiration specimens fail to yield diagnostic value, then open biopsy is recommended. All samples should be sent for culture, smear, and histology examinations (Wood and Miedzinski, 1996). Culture is the gold standard diagnosis (Wood and Miedzinski, 1996). After staining with India ink, the organism resembles cells with a halo due to a lack of capsule staining, and it is easily detected using specific PAS, mucicarmine, and GMS stains (although it is poorly stained by H & E). Urease-positive mucoid colonies are produced in cultures on SDA agar usually within 3–5 days (Mitchell and Perfect, 1995; Qadir et al., 2011; Jain et al., 2013). Currently available commercial methods for yeast identification, such as API 20 AUX (bioMerieux, Paris, France) and Vitek (bioMerieux), are used to identify the yeast-like organisms (Qadir et al., 2011; Zhou et al., 2013). Once the organisms are detected, identifying the strain is recommended as mentioned above.

Examinations for disseminated cryptococcosis should be performed after identification. Relevant examinations generally consist of the following procedures: lumbar puncture for antigen testing and culture, blood culture, urine culture, sputum culture, and skin lesion culture (Wood and Miedzinski, 1996). Recently, a marrow aspirate was considered in the diagnosis of disseminated cryptococcosis (Venkatachala et al., 2010). Testing for serum cryptococcal antigen using a latex agglutination test (LA), an enzyme immunoassay (EIA) or lateral flow assay (LFA) is useful for diagnosis given their sensitivity and specificity (Bruno et al., 2002; Lindsley et al., 2011; Hansen et al., 2013). However, serum cryptococcal antigen is not always positive even when infection is demonstrated via culture (Hawkins and Flaherty, 2007). CSF cryptococcal antigen testing is more highly sensitive and specific for meningitis than serum cryptococcal antigen testing (Hawkins and Flaherty, 2007).

The radiological findings of skeletal cryptococcus were non-specific (Chleboun and Nade, 1977; Behrman et al., 1990; Wood and Miedzinski, 1996); sclerosis or periosteal reaction, which are typical symptoms associated with tumors, were found in our study (Levine et al., 1985; Bosch et al., 1994; Witte et al., 2000; McGuire et al., 2011). Furthermore, patients with poor immune status were less likely to show radiological features of malignancy. The differential diagnoses based on radiological features included microbial infections, namely Staphylococcus aureus, Brucella, Actinomyces, tuberculosis, and neoplasms such as Ewing's sarcoma, osteogenic sarcoma, enchondroma, and giant cell tumor (Behrman et al., 1990; Witte et al., 2000). In addition, the diagnosis of osteomyelitis is commonly indicated based on radiological studies that are non-specific for *C neoformans* (Behrman et al., 1990), and our results showed that patients can contract osteomyelitis regardless of the presence of immune abnormalities. Radiological studies should be routinely performed because they assist the final diagnosis and can be used as a monitoring index to detect the efficacy of therapy based on radiological improvement, healing, or resolution.

The insidious course of this disease contributes to the delays in diagnosis (Bunning and Barth, 1984; Matsushita and Suzuki, 1985; Baldwin et al., 1988). Importantly, tuberculosis was the most common reason for misdiagnosis. Although our results indicated that delayed diagnosis did not contribute to a worse survival rate, clinicians must be alerted to this disease and identify it in a timely manner.

Except for the lungs ans CNS, no standardized treatment protocol exists for cryptococcal infection for specific body sites (Jain et al., 2013; Ramkillawan et al., 2013). For these sites, surgery in conjunction with antifungals, antifungals alone, or (rarely) surgery alone have been demonstrated to be effective. According to the Infectious Disease Society of America (IDSA), surgery, which effectively and rapidly eliminates the fungal burden and prevents the contiguous spread of infection (Chleboun and Nade, 1977; Govender et al., 1988; McGuire et al., 2011), should be performed to patients with persistent or refractory bone disease (Perfect et al., 2010). Surgery also provides physicians with the opportunity to obtain specimens for histological and microbiological examination to make a definitive diagnosis (Ramkillawan et al., 2013). The selection of antifungal agents and the duration of therapy depends on factors including disease severity, host immune status, the infection site, and therapeutic response (Qadir et al., 2011; Zhang et al., 2012). Systemic therapy consists of AMB, 5-FC, fluconazole, ketoconazole, or some combination therein (Bryan, 1977; Galloway and Schochet, 1981; Stead et al., 1988; Ueda et al., 1992; Perfect et al., 2010). Although, combination therapy with AMB and 5-FC (with or without surgery) did not outperform AMB alone (with or without surgery) in terms of improving the mortality rate (which might be due to the small number of cases), combined therapy is recommended. This result is contrary to previous reports (Bryan, 1977; Poliner et al., 1979; Shaff et al., 1982; Raftopoulos et al., 1998; Perfect et al., 2010) and might be due to the small number of cases reviewed here. Thus, combined therapy is recommended given the prevention of secondary drug resistance, the shorter duration of therapy, smaller total dosage, and the reduced likelihood of side effects (Bryan, 1977; Raftopoulos et al., 1998; Jain et al., 2011). The most common treatment is a combination of AMB and 5-FC, which can decrease the high nephro- and hepatotoxicity of AMB (Bruno et al., 2002). The lipid formulation of AMB is used in patients with renal impairments (Perfect et al., 2010). The IDSA indicates that 200-400 mg per day of oral fluconazole for 6–12 months is the treatment of choice for patients with immunocompetent status and non-meningeal, non-pulmonary cryptococcosis because of its significantly reduced toxicity (Agadi et al., 2010; Perfect et al., 2010; Qadir et al., 2011; Zhou et al., 2013). Several case reports published over the last decade have demonstrated the successful treatment of cryptococcal osteomyelitis using fluconazole alone (Hummel et al., 1996; Wildstein et al., 2005; Agadi et al., 2010; Qadir et al., 2011; Zhou et al., 2013). Patients with disseminated cryptococcosis had unfavorable outcomes in our study, and this result is consistent with previous reports (Behrman et al., 1990; Bruno et al., 2002; Hawkins and Flaherty, 2007). Combination induction therapy of AMB and 5-FC followed by consolidation and maintenance therapies with fluconazole are recommended for patients with disseminated cryptococcosis (Perfect et al., 2010; Zhang et al., 2012). Suppressive treatments for disseminated disease due to CNVG are the same as those for CNVN described above. The ultimate duration of therapy is unknown, but it should be based on clinical findings, ESRs, serum cryptococcal antigen levels, and radiological improvements (Goldshteyn et al., 2006; Zhang et al., 2012).

The outcomes of patients with disseminated cryptococcosis were unfavorable, and those of patients with or without immune abnormalities were similar; these findings differ from previous studies (Corral et al., 2011; Jou et al., 2011; Qadir et al., 2011; Jain et al., 2013). This disparity might be explained by the recent CNVG outbreak. The recurrence rate of skeletal cryptococcosis is low (Hawkins and Flaherty, 2007). However, unlike the successful treatment of cryptococcal meningoencephalitis (demonstrated via CSF culture) and that of pulmonary cryptococcosis (demonstrated via sputum culture or the specimens obtained during bronchoscopy) (Perfect et al., 2010), it is difficult to prove the success of primary therapy in skeletal cryptococcus. Hence, once cannot distinguish relapse from recurrence. Clinical and radiographical follow-up assessments, as well as serum cryptococcal antigens, should be monitored carefully. For the qualitative or quantitative detection of serum cryptococcal antigen, a latex agglutination test (LA), an enzyme immunoassay (EIA) or a LFA should be used, and LFA shows excellent overall agreement with EIA (Lindsley et al., 2011; Hansen et al., 2013). Once an abnormal manifestation occurs during the primary therapy, a larger total dosage is recommended. If the abnormal manifestation recurs, then susceptibility testing should be performed to formulate the best therapy by evaluating the changes in the minimum inhibitory concentration (MIC) of the recurrent isolates and original isolates (Perfect et al., 2010). Prednisone prescribed for other diseases should be tapered during skeletal cryptococcosis treatment, given the drug's effect on immunity (Noh et al., 1999).

## Concluding remarks

Skeletal cryptococcosis occurs in patients with immune abnormalities and even in those who are immunocompetent. An immune abnormality is a risk factor but it does not predict mortality. Likewise, neither immunocompetence nor immune abnormalities predicted the deaths caused by recent CNVG outbreaks. Patients with (especially classic immunodeficiencies) or without immune abnormalities present with dissemination, and these patients are more likely to have unfavorable prognoses. Clinicians must be alert to this disease and be able to identify the particular fungal strain. No standardized treatment protocol exists for skeletal cryptococcosis. Although, combination therapy with AMB and 5-FC (with or without surgery) did not outperform AMB alone (with or without surgery) in terms of improving the mortality rate (which might be due to the small number of cases reported), combined therapy is recommended. Given that our series was unable to collect all information (which led to difficulties in further elucidating this disease), creating a disease database of skeletal cryptococcosis is recommended.

## Author contributions

Heng-Xing Zhou and Lu Lu, as first coauthors, contributed equally to drafting and revising the review with input from all authors. All authors approved the final version.

### Conflict of interest statement

The authors declare that the research was conducted in the absence of any commercial or financial relationships that could be construed as a potential conflict of interest.
